# Study of the Effect of Methyldiethanolamine Initiator on the Recording Properties of Acrylamide Based Photopolymer

**DOI:** 10.3390/polym12040734

**Published:** 2020-03-25

**Authors:** Brian Rogers, Suzanne Martin, Izabela Naydenova

**Affiliations:** 1 Centre for Industrial and Engineering Optics/School of Physics and Clinical and Optometric Sciences, College of Sciences and Health, Technological University Dublin, Kevin Street, D08 NF82 Dublin, Ireland; c14507363@mytudublin.ie (B.R.); suzanne.martin@tudublin.ie (S.M.); 2 FOCAS Institute, Technological University Dublin, 13 Camden row, D08 CKP1 Dublin, Ireland

**Keywords:** photopolymers, holography, holographic recording materials, diffraction gratings

## Abstract

The use of Holographic Optical Elements (HOEs) in applications, such as in light shaping and redirection, requires certain characteristics such as a high Diffraction Efficiency, low angular selectivity and stability against UV damage. In order to maximize the performance of the HOEs, photosensitive materials are needed that have been optimised for the characteristics that are of particular importance in that application. At the core of the performance of these devices is the refractive index modulation created during holographic recording. Typically, a higher refractive index modulation will enable greater light Diffraction Efficiency and also operation with thinner devices, which in turn decreases the angular selectivity and the stability of the refractive index modulation introduced during recording, which is key to the longevity of the device. Solar concentrators based on volume HOEs can particularly benefit from thinner devices, because, for a solar concentrator to have a high angular working range, thinner photopolymer layers with a smaller angular selectivity are required. This paper presents an optimisation of an acrylamide-based photopolymer formulation for an improved refractive index modulation and recording speed. This was achieved by studying the effect of the concentration of acrylamide and the influence of different initiators in the photopolymer composition on the diffraction efficiency of holographic gratings. Two initiators of different molecular weights were compared: triethanolamine (TEA) and methyldiethanolamine (MDEA). A fivefold increase in the rate of grating formation was achieved through the modification of the acrylamide concentration alone, and it was also found that holograms recorded with MDEA as the initiator performed the best and recorded up to 25% faster than a TEA-based photopolymer. Finally, tests were carried out on the stability of the protected and unprotected photopolymer layers when subjected to UV light. The properties exhibited by this photopolymer composition make it a promising material for the production of optical elements and suitable for use in applications requiring prolonged exposure to UV light when protected by a thin melinex cover.

## 1. Introduction

Photopolymers have been explored for many applications and one of them is as a media for holographic recording [1]. As their development continues it is becoming clear that their properties are also well suited to the development of a variety of different devices [2,3,4]. These properties include a long shelf life, high diffraction efficiency and self-development [5]. High refractive index modulation is an important characteristic for many holographic devices, especially when optimising the diffraction efficiency of devices recorded in thinner layers. Examples include holographic optical elements (HOEs) requiring a large angle of operation, such as those designed for solar concentration and light dispersion because reducing layer thickness increases angular acceptance [2]. In the opposite case, in holographic data storage applications, thicker devices are preferred, since the high-angular selectivity makes it possible to achieve higher data density [3].

The initiating/sensitising system facilitating the photopolymerisation process has been a subject of much study [6,7,8,9]. Multiple methods of materials’ sensitisation and the initiation of polymerisation reaction exist. Systems involving the interaction of a dye molecule with an initiator in order to produce a free radical capable of interacting with a monomer are an example of one such system [9,10]. Some molecules can act as both a dye and an initiator which can change reaction rates between the initiator and monomer [11]. Improvements in the sensitivity of photopolymer materials can be driven by changes to the dye/initiator system. The rates of reaction and absorption spectrum are two of the main properties affected by this system [6,7,8,9,12]. 

**Figure 1:**
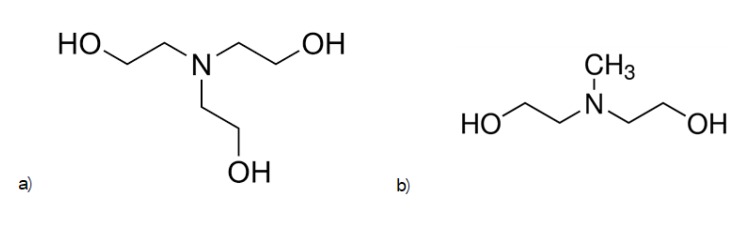
Structure of the studied initiators. (**a**) triethanolamine (TEA) and (**b**) methyldiethanolamine (MDEA).

The rate of fabrication of HOEs is an important factor when considering the mass production of photopolymer-based holographic devices. Higher production rates and lower costs are some of the main advantages of faster initiation and recording. Altering the initiator system can have an effect on the recording time. It has been previously observed that different initiators are capable of changing the bleaching rate of photopolymers implying different reaction rates [9]. Guoqiang et al. found that methyldiethanolamine (MDEA) had the fastest bleaching time of a number of initiators, among them triethanolamine (TEA) and 1,4-Diazabicyclo[2.2.2]octane (DABCO). In dye-sensitised photopolymers based on free radical chain polymerization, the creation of a free radical is, most of the time, a process relying on the interaction of at least two molecules, the absorbing dye and the initiator. Thus, the size of these molecules is expected to be an important factor in the rate of radical generation. Two different initiators, TEA and MDEA, are used in this study, which have similar chemical structures, but different sizes (Figure 1). A third molecule, diethylmethanolamine (DMEA), was used, however, layers made with this molecule dried poorly, likely due to the high vapour pressure.

The lifetime of the recorded holographic devices when subjected to conditions of high temperature and UV exposure must also be considered, especially when, during their use, these devices are under constant solar exposure, as in the case of solar concentrators. Callados et al. present a series of solar concentrating holographic devices [2] many of these devices are intended to function within Earth’s atmosphere where they will be subject to UV radiation in the order of 50 W. Some holographic concentrating devices have the potential to work in tandem with photovoltaic (PV )cells on satellites, where they will be subject to UVB and UVC radiation [13]. The chemical composition of the photopolymer can have effects on the stability and pre- or post-recording shelf life of the device. Rajesh et al., for example, report an improvement in the pre-recording shelf life of the material with the addition of a sensitising dye [5]. The stability of any new developed photopolymer formulations must be tested with respect to the envisaged conditions of operation of the device.

Acrylamide is a popular monomer used in many photopolymer formulations and is a major contributor to the achieved refractive index modulation in the photopolymer. Higher concentrations of monomer have shown to increase reaction rates as well as refractive index modulation [14]. This study utilises a photopolymer composition with an optimised concentration of acrylamide for maximum refractive index modulation while maintaining the optical quality of dry layers (too high a monomer concentration in the layer can lead to its crystallization on the layer surface). The stability of the TEA- and MDEA-based photopolymer is also compared through UV exposure tests. 

We have recently reported [15] results on optimisation of the monomer concentration for high refractive index modulation in acrylamide-based photopolymer utilising a Triethanolamine (TEA) as an initiator. An increase in the acrylamide concentration of 66% resulted in a 50% higher refractive index modulation with values reaching 0.005 in 40 micron layers. The rate of formation of the holographic gratings in layers of thickness varying between 20–45 μm was also studied for two-initiators, TEA and methyldiethanolamine (MDEA), and it was observed that it increases by a factor of 2 when MDEA is used instead of TEA. When MDEA and TEA are compared at different thicknesses, MDEA was shown to out-perform TEA as an initiator at a layer thickness in the order of 45 μm.

Mathematical models have shown that the background refractive index has a significant effect on the overall refractive index modulation achieved by a holographic recording of the photopolymer system [16]. This background refractive index can be affected by the binder refractive index and initiator molecule used in free radical generation, since they are present in equally large quantities. The two initiators used in this study have different refractive indices (1.4852 for TEA and 1.4685 for MDEA), potentially changing the overall background refractive index, and thus the refractive index modulation achieved.

Other methods, such as the introduction of nanoparticles, have proven to be an effective method for increasing refractive index modulation, decreasing shrinkage and adding stability to photopolymer compositions, but it increases the photopolymer cost significantly [17,18].

This paper further investigates increases in the refractive index modulation as well as the rate at which the refractive index modulation is created in the material, though optimization of the monomer content and the use of an alternative initiators. The robustness of the gratings recorded in the new formulation is tested through exposure to UV and compared. 

## 2. Theoretical Background

### 2.1. Diffraction Efficiency of Thick Volume Gratings

The refractive index modulation in volume phase holographic gratings with a given diffraction efficiency *η* can be calculated using Kogelnik’s theory [19] by utilising the equation below.

(1) $$\Delta n=\frac{\sin^{-1}\sqrt{\eta \;}\cos\left(\theta \right)\lambda \;}{\pi T}$$

where Δn is the refractive index modulation, θ is the angle of incidence of the probe laser beams inside the medium, *λ* is the wavelength of the probing laser, *T* is the thickness of the photopolymer layer and η is the diffraction efficiency.

The thickness of the gratings can be approximated from the Bragg selectivity curves using the equation below

(2) $$T=\frac{0.87d}{\Delta \theta }$$

where 2Δ*θ* is difference in angle between the first two minima on both sides of the Bragg curve peak, and d is the period of the grating.

The period of the grating is determined by the angle between the two recording beams 2*θ* and the wavelength of the recording light. It can be determined by the following equation

Λ = *λ*/2sin*θ* (3)

### 2.2. Dependence of the Angular and Wavelength Selectivity on the Thickness of Gratings

Holograms will diffract different amounts of light depending on the angle and the wavelength of the incident light beam. The dependency of diffraction efficiency on angle/wavelength near the peak of Bragg diffraction is referred to as a Bragg curve. A hologram has a high angular/wavelength selectivity when the range of angles/wavelengths between the first minima on either side of the Bragg curve is narrow. In the design of solar concentrators, a broad angular/wavelength selectivity is needed so that more light can be collected onto the solar cell.

**Figure 2:**
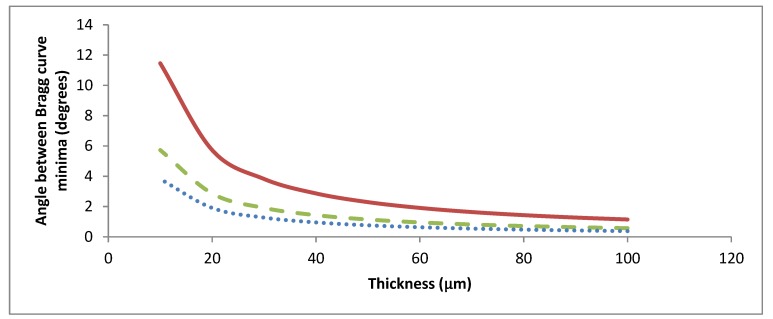
Angle between Bragg curve minima vs. hologram thickness at 500 (solid red), 1000 (dashed green) and 1500 (dotted blue) lines/mm.

Figure 2 shows the effect of the thickness of the hologram on the angular selectivity at different spatial frequencies. Lower spatial frequencies and thicknesses decrease the angular selectivity. Having a low angular selectivity is required for some solar concentrating devices, as it removes the need for solar tracking systems.

Wavelengths will diffract with different efficiencies at the Bragg angle. Photovoltaic cells work most efficiently in certain wavelength ranges; for the most efficient system it is important that useful wavelengths are diffracted with the most efficiency. Ghosh et al. [20] extensively models how the diffracted wavelength is affected by conditions like spatial frequency and thickness. 

### 2.3. Expected Effect of the Size of the Initiator

In the photopolymer system studied here, the diffusion of molecules plays a very important role, since, during exposure to structure light field (such as an interference pattern) a concentration gradient of the monomer and polymer molecules is created between the bright and the dark areas. This drives mass transport of the monomer molecules from dark to bright, and of mobile polymer molecules from bright to dark, regions. Diffusion is also important in the process of the creation of free radicals, since the system requires the interaction of two molecules—the light absorbing dye and the electron donor. In order to estimate how molecules diffuse throughout the photopolymer, the Stokes equation is used to find the ratio of the drag force of MDEA to TEA. The Stokes equation gives the frictional force that a molecule experiences when moving in a fluid environment [21]

(4) F=6πaη

where *F* is the drag force, *a* is the molecule’s radius (assumed to be a sphere) and η is the viscosity of the solvent. The purpose of this equation is to illustrate the fact that molecular volume is a factor to be considered when determining molecular diffusion. According to this model, by calculating the volume of both TEA and MDEA, and assuming them to be a sphere, TEA should experience a drag force 1.4 times greater than MDEA. In reality, especially in the case of photopolymers, there are a number of additional factors that will affect the diffusion. These include temperature and humidity, which will affect the permeability of the layer, viscosity of both the solvent and the diffusing molecule, and the photopolymer layer thickness (for layers with low thickness, in the order of a micrometre).

## 3. Materials and Methods 

### 3.1. Effect of Acrylamide Concentration on Growth Rate

A PVA solution (9 wt %) was prepared by dissolving 10 g of PVA in 100 mL of deionized water under constant stirring at 50 °C. The solution was then poured through filter paper. A dye stock solution was also prepared by dissolving 0.11 g of Erythrosine B in 100 mL of deionized water. 

**Figure 3:**
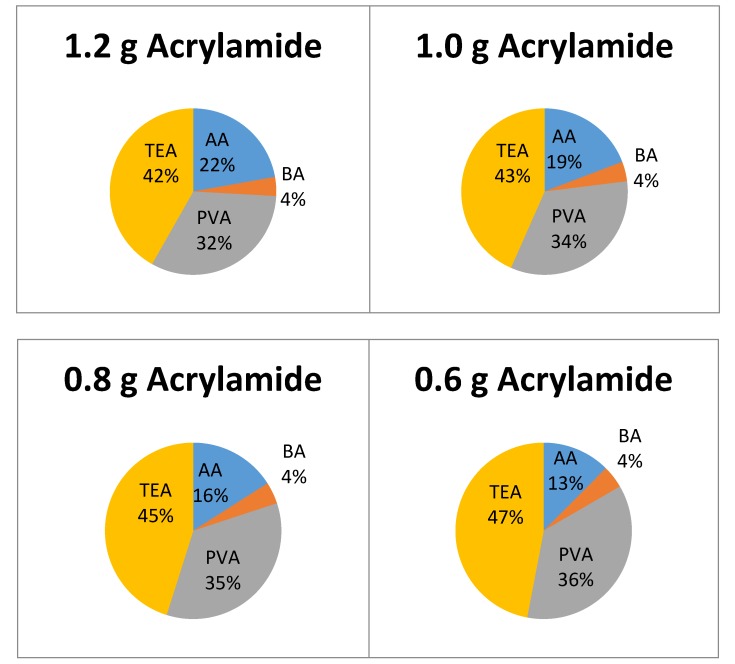
Mass ratios of photopolymer with varying acrylamide concentration. AA—acrylamide, BA-N,N’—Methylenebisacrylamide, TEA—Triethanolamine, PVA—Polyvinylalcohol.

The photopolymer was prepared by mixing 4.38 mL of the PVA solution, 1 mL of the dye solution, 0.5 mL of TEA initiator, 0.05 g of bis-acrylamide cross linker and 0.25 g of acrylamide. Three other acrylamide masses (0.15, 0.20, 0.30 g) were also used, in order to determine its effect on growth rate. The composition of the dried layers is shown in Figure 3.

A pipette was used to spread 0.500 mL of photopolymer onto glass microscope slides with an area of 76 by 26 mm^2^. The samples were left to dry overnight.

### 3.2. Preparation of Samples with Different Initiators

**Figure 4:**
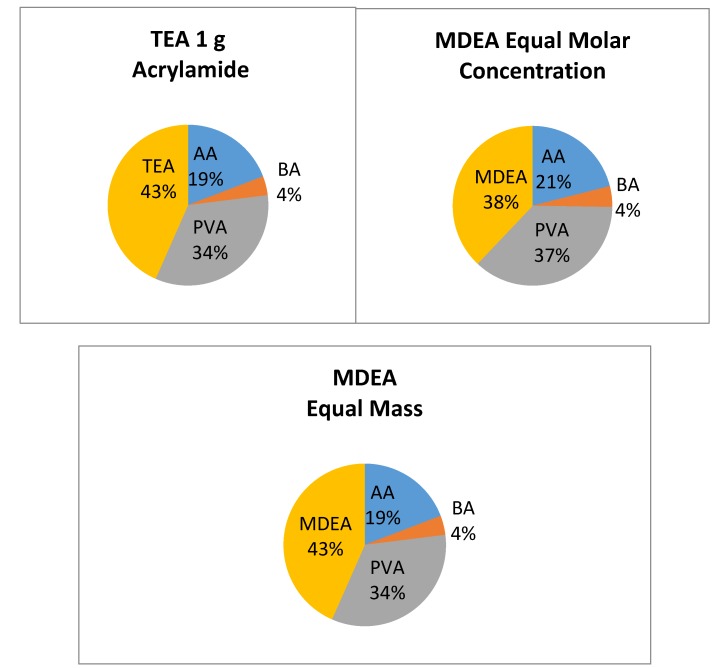
Mass distribution in the solid photopolymer layer. AA—acrylamide, BA-N,N’—Methylenebisacrylamide, TEA—Triethanolamine, MDEA—Methyldiethanolamine, PVA—Polyvinylalcohol.

For the initiator tests, the photopolymer was prepared following the procedure outlined above, using an acrylamide mass of 0.25 g. Two sets of MDEA samples were prepared—the first set having the same molar concentration as in the TEA samples, and the second set having the same mass of MDEA as in the TEA samples. For the preparation of the first set, 0.430 mL of MDEA was used. For the second set, with equal amounts of initiator by mass, 0.543 mL of MDEA was used. The mass distribution of these solutions can be seen in Figure 4, below. Both types of sample were studied in order to identify if any effect of decreasing the initiator overall content in the layer was observed. It is known that the initiator in this photopolymer system also plays the role of a plasticizer. As seen in Figure 4, the amount of initiator in the MDEA sample with the same molar concentration is decreased by 12%, for that reason we have the second set, where the percentage of the initiator (and plasticizer) in the layer for the TEA and MDEA samples is identical. 

The samples were prepared identically to the previous section, using a pipette to spread 0.500 mL of solution onto a microscope slide.

### 3.3. Recording

**Figure 5:**
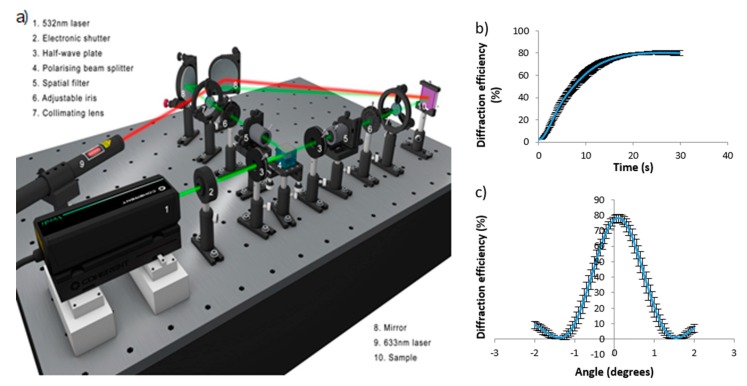
(**a**) Holographic recording (recording beams are shown in green) and real-time monitoring (probe beam are in red) set up; (**b**) Example average growth curve for four samples prepared with 0.592 M acrylamide photopolymer solution; (**c**) Example Bragg curve (bottom).

The set up in Figure 5 was used to record transmission holographic gratings in the samples. After opening a shutter, the 532 nm beam passes through a half-wave plate, followed by a polarising beam splitter. One beam is spatially filtered, collimated and directed towards the sample using a mirror. The other beam travels through another half-wave plate, then is spatially filtered and collimated. This system allows the intensity of both beams to be controlled and made equal. The two beams meet at the sample where the holographic recording takes place. The 633 nm beam is on Bragg and goes on to a power meter, where the grating formation is tracked in real time using a computer. The angle between the two recording beams was 24.6°, resulting in an interference pattern with a special frequency of 800 lines/mm. The combined beam intensity is approximately 0.5 mW/cm^2^. The beams had a diameter of 1 cm. After the samples were recorded, a Bragg curve of the holographic grating was taken using the rotating stage on which the sample was placed. An example real-time diffraction efficiency growth curve and a Bragg curve are shown in Figure 5b,c, accordingly. These curves are average measurements during recording in four separate layers, and the size of the error bars for ach point on the curves demonstrates the excellent repeatability of the experiment.

To calculate the refractive index modulation, first the grating thickness must be determined. This information can be extracted from the Bragg curve of a hologram. Figure 5c shows an example Bragg curve for a given acrylamide concentration. Four holograms were recorded for each concentration of acrylamide. 

The difference between the minima was found using these Bragg curves in order to calculate the layer thickness. Kogelnik’s equation was then used to find the refractive index modulation for each sample. This is important as it allows us to account for small deviations in the layer thickness and their contribution to diffraction efficiency. The thickness of the samples lay in the range of 36–47µm. These deviations are likely caused by inconsistencies in the hand coating and drying process. This effect can be particularly exaggerated at the edges of the sample; care was taken to record only in the middle of the samples.

### 3.4. UV Stability Tests

A comparison between the stability of TEA- and MDEA-based photopolymer was carried out. Six samples were prepared, three with TEA as initiator, and three with MDEA as initiator. The concentration of initiator was constant in both sets of samples. In this case, 0.300 mL of each photopolymer was spread on the glass slides. This was carried out in order to keep the layers thin and avoid over-modulation when bleaching. On each sample, three holograms were recorded using the set up in Figure 5. After the holograms were recorded the samples were bleached for 6 min using a DYMAX ECE series UV light curing flood lamp. A Bragg curve of every sample was taken before and after bleaching. Some of the samples were covered with melinex and silicone oxide-coated melinex, so that their degradation over time could be compared. After covering, the samples were placed in the UV curing system again for 40 h. The UVA intensity was 60 mW/cm^2^. The Bragg curves were taken again after 40 h, and their stability was evaluated by calculating a robustness factor, the ratio of the post-UV exposure value of the refractive index modulation to the original value of refractive index modulation.

## 4. Results

### 4.1. Effect of Monomer Concentration on Initial Rate of Recording

The rate of growth in the diffraction efficiency during exposure was measured for each acrylamide concentration. For comparison purposes, the initial change of diffraction efficiency with time was determined from the real-time grating growth curve; an example curve is presented in Figure 5b.

**Figure 6:**
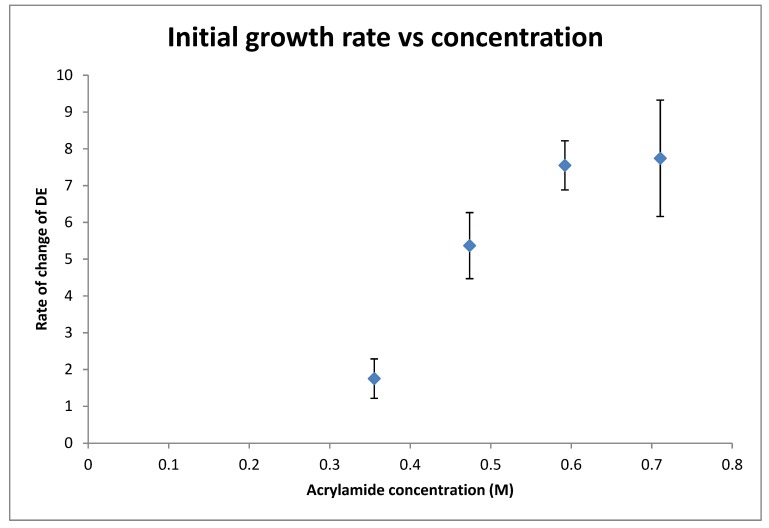
Initial growth rate vs. acrylamide concentration.

Four samples were recorded for each concentration. The first 5 s of the curve can be approximated as a straight line, and the initial growth rate was determined by finding the slope of this section. Figure 6 shows the growth rate for each concentration. The size of the error bars give a sense of the stability and repeatability of the growth curves.

The growth rate increases non-linearly with acrylamide concentration. The growth rate appears to plateau at an acrylamide concentration of 0.592 M, after which increasing the concentration results in no further growth. The consistent growth curves and high refractive index modulation made the 0.592 M solution the ideal concentration for further tests with the different initiators.

### 4.2. Study of the Effect of the Initiator on the Recording Properties of the Photopolymer

After the optimum acrylamide concentration was determined, the TEA initiator was compared with the MDEA initiator.

**Figure 7:**
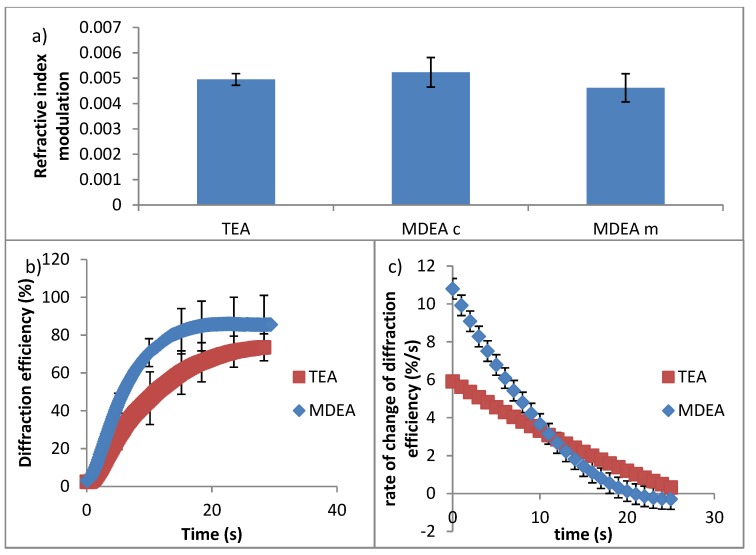
(**a**) Refractive index modulation for each initiator composition. TEA, MDEA (same molar concentration), MDEA (same mass), from left to right (top) (**b**) Growth curves (bottom left) and (**c**) their rate of change of diffraction efficiency for TEA and MDEA with the same molar concentration (bottom right).

Growth and Bragg curves were recorded for each initiator composition; the thickness, refractive index modulation and growth rates were determined identically to the previous section. The refractive index modulation for each initiator is shown in Figure 7.

As seen in Figure 7a, the TEA and MDEA performed similarly in terms of refractive index modulation. Any difference in the performance of three sets of samples is within the experimental uncertainty.

The growth rate of the TEA and MDEA compositions is compared in Figure 7c. The rate of change in diffraction efficiency was calculated by finding the derivative of a polynomial fit to the growth curves. The MDEA compositions had larger initial growth rates than the TEA composition. MDEA holograms reach saturation before the TEA, as seen in Figure 7b. Reaching saturation sooner causes the growth rate of the MDEA to slow below that of TEA, reaching zero, while TEA holograms still experience growth. The MDEA composition with the same molar concentration as TEA and the composition with the same mass of TEA had similar growth rates. This higher growth can likely be attributed to two things: molecule size and number of hydrogen bonds. MDEA is a smaller molecule; this could make diffusion through the polymer matrix easier. MDEA also has one less alcohol functional group than TEA; this reduces the number of potential hydrogen bonds it could form in the matrix, which would otherwise slow the rate of diffusion. 

### 4.3. Investigation of the Effect of Exposure to UV Light on the Diffraction Efficiency of the Diffraction Gratings

**Figure 8:**
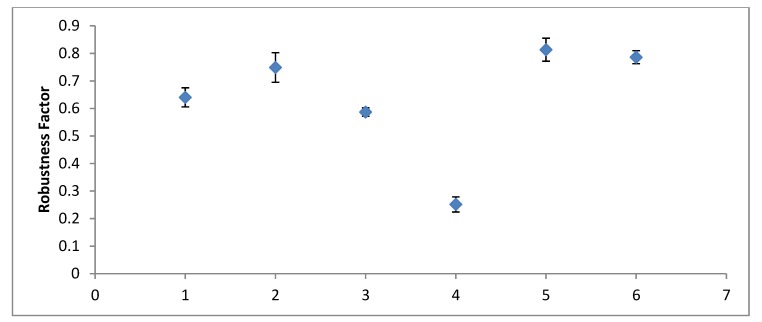
The degree of robustness of each set of samples. 1—TEA Uncovered, 2—TEA Melinex, 3—TEA UV Protection, 4—MDEA Uncovered, 5—MDEA Melinex, 6—MDEA UV Protection.

For each set of samples, the refractive index modulation was calculated before and after degradation. The robustness factor is the ratio of the post-UV exposure value of the refractive index modulation to the original value of refractive index modulation. The degradation factor was determined for each sample and can be compared in Figure 8 below.

In general, samples coated with melinex degraded less than other samples, with both the TEA- and MDEA-based samples degrading to between 0.7 and 0.85 of their original values. The UV protective plastic performed no better than the melinex. The uncovered TEA-based sample performed within a similar range to the coated samples. The uncovered MDEA sample, however, degraded almost completely. In each case, this was repeated for three samples.

## 5. Discussion

The research conducted confirms that variations in the concentration of acrylamide drastically affect the properties of the photopolymer and optimises it for this formulation. Both the refractive index modulation [1] and the time to record can be improved by working with an optimal amount of acrylamide. Using an acrylamide concentration of 0.592 M, the largest refractive index modulation achieved in the TEA-initiated photopolymer was 6.8 × 10^−3^. On average, the refractive index modulation was increased by 50% compared to compositions containing 0.6 times less acrylamide. The rate of grating formation was increased by 4.3 at higher acrylamide concentrations.

MDEA, which has a similar chemical composition to TEA, was found to further improve the recording time of holograms. MDEA showed a 25% decrease in recording times compared to the photopolymer initiated with TEA. The increased recording rate in the MDEA-based photopolymer compared to the TEA-based photopolymer is likely due to its smaller molecular size, which enables faster mass transport.

When subjected to UV radiation, both MDEA and TEA holograms were found to degrade to some degree. Uncovered MDEA-initiated samples underwent the largest decrease in refractive index modulation, reducing their diffraction efficiency to 0.25 of their original value post-exposure. The covered MDEA samples, however, were some of the best-performing samples. This large difference in the performance of covered and uncovered samples was not observed with the TEA samples.

## 6. Conclusions

A photopolymer with an increased dynamic range and sensitivity has been developed. Increased acrylamide concentrations combined with an MDEA initiator has led to higher refractive index modulation and holograms recorded in a shorter time period compared with photopolymer using TEA as an initiator and lower acrylamide concentrations. When appropriately coated, degradation in the presence of 60 mW/cm^2^ UVA radiation for 40 h was kept under 25% by covering the samples with melinex.
